# Use of glucocorticoids in the management of immunotherapy‐related adverse effects

**DOI:** 10.1111/1759-7714.13589

**Published:** 2020-09-06

**Authors:** Hanping Wang, Jiaxin Zhou, Xiaoxiao Guo, Yue Li, Lian Duan, Xiaoyan SI, Li Zhang

**Affiliations:** ^1^ Department of Respiratory Medicine, Peking Union Medical College Hospital Peking Union Medical College and Chinese Academy of Medical Sciences Beijing China; ^2^ Department of Rheumatism and Immunology, Peking Union Medical College Hospital Peking Union Medical College and Chinese Academy of Medical Sciences Beijing China; ^3^ Department of Cardiology, Peking Union Medical College Hospital Peking Union Medical College and Chinese Academy of Medical Sciences Beijing China; ^4^ Department of Digestive Medicine, Peking Union Medical College Hospital Peking Union Medical College and Chinese Academy of Medical Sciences Beijing China; ^5^ Department of Endocrinology, Peking Union Medical College Hospital Peking Union Medical College and Chinese Academy of Medical Sciences Beijing China

**Keywords:** Glucocorticoids, glucocorticosteroids, immune checkpoint inhibitor, immune suppressors, immunotherapy‐related adverse effects

## Abstract

Immune checkpoint inhibitors (ICIs) activate host antitumor immunity to kill tumor cells. However, ICI therapy may be accompanied by a series of immunotherapy‐related adverse effects (irAEs) caused by activated autoreactive T cells. Glucocorticoids are the mainstream therapy for irAEs. However, the usage, dosage and course of treatment of irAEs with glucocorticoids differs from those used in classic autoimmune diseases. Furthermore, the long‐term use of large doses of glucocorticoids may cause serious adverse effects. In this article, the mechanism, dosage forms, adverse effects and management of glucocorticoids are described in detail, providing references and suggestions for oncologists to use glucocorticoids in the treatment of irAEs.

## Abstract 2

Glucocorticoids are the mainstream therapy for irAEs. We described the mechanism, dosage forms, adverse effects and management of glucocorticoids. We aimed to provide references and suggestions for oncologists to use glucocorticoids in the proper treatment of irAEs.

Immune checkpoint inhibitors (ICIs) can specifically bind to the immune checkpoint receptors on T cells and cancel their inhibitory effect, leading to the activation of an antitumor immune response. However, these activated T cells can also attack normal tissues of the human body, causing ICI‐related toxicities. These toxicities can affect various parts of the body, causing a new type of autoimmune disease, known as immunotherapy‐related adverse effects (irAEs).^1^ The basic strategy to treat irAEs is immunosuppressive therapy. Glucocorticosteroids (GCS) are used in the treatment of irAEs because they powerfully suppress the immune system, thus rapidly inhibiting the inflammation. However, GCS usage, dosage, and course of treatment in the management of irAEs differ from those in the treatment of classic autoimmune diseases.

In the 1930s, members of different research groups isolated the inactive form of the glucocorticoid cortisol (cortisone) and elucidated how cortisol can be artificially synthesized. The use of glucocorticoids in clinical practice grew gradually throughout the following decade. The newly synthesized glucocorticoids from 1950 to 1960, including prednisone and methylprednisolone, have stronger anti‐inflammatory and immunosuppressive effects and fewer side effects than mineralocorticoids, which have been previously widely used in clinical practice.^2^ However, the long‐term use of large doses of glucocorticoids is still associated with serious adverse reactions that affect various organs and systems including the endocrine system, bone, skin, muscle, and eyes. In addition, the long‐term use of large doses of glucocorticoids is often accompanied by opportunistic infections, and may even lead to death. This article will describe in detail the action mechanism, dosage form, dosage, and adverse effects, providing references and suggestions for oncologists to use glucocorticoids in clinical practice.

## Molecular mechanisms of glucocorticoids

The different molecular mechanisms of glucocorticoids provide the basis for optimizing their applications in clinical practice.

The molecular mechanisms of glucocorticoids can be divided into genomic mechanisms and nongenomic mechanisms.^3^, ^4^ The classical genomic mechanism is mediated by cytosolic glucocorticoid receptor alpha (cGCRa), and the nongenomic mechanisms can be further divided into three types: (i) cGCR‐mediated nongenomic mechanism; (ii) specific nongenomic mechanism (eg, physicochemical interactions with plasma membrane at high glucocorticoid concentrations); and (iii) membrane GCR‐mediated mechanism.^4^

### Classical cGCR‐mediated genomic mechanism

The cGCR is a multiprotein molecule expressed in most cells of the body. The cGCR contains several heat shock proteins (Hsp) including Hsp90, Hsp70, Hsp56, and Hsp40. The cGCR can also interact with immunoaffinity proteins, chaperones (such as p23 and Src), and mitochondrial activated protein kinase (MAPK) signal pathway kinases. Glucocorticoids are a class of GCS. Glucocorticoids are lipid‐soluble and diffuse readily across cell membranes. When glucocorticoids bind to the cGCR, the activated glucocorticoid‐cGCR complexes participate in the regulation of gene transcription. At the same time, chaperone proteins attached to the cGCR enter the cytoplasm and 20 minutes after binding, the activated glucocorticoid‐cGCR complexes translocate into the nucleus. The binding of glucocorticoid‐cGCR complexes to specific DNA‐binding sites affects the transcription and translation processes in the nucleus. Some genes are upregulated to promote the synthesis of anti‐inflammatory proteins, while some genes responsible for the production of proinflammatory mediators are downregulated to achieve immunosuppression. This significant increase in anti‐inflammatory protein production takes more than 30 minutes, and it usually takes hours or days for GCS to diffuse across the cell membranes, bind to cGCR, transport the glucocorticoid‐cGCR complexes into nuclei, combine with the promoter region of target genes, and initiate transcription and translation until new proteins are produced.^3^ Therefore, the rapid anti‐inflammatory and antiallergic effects of GCS cannot be fully explained by classical genomic mechanisms.

### **Nongenomic mechanisms of** **GCS**

Three different nongenomic mechanisms may explain the rapid anti‐inflammatory and immunosuppressive effects of GCS.^5^

#### Nonspecific interaction between GCS and cell membrane

Large doses of GCS can directly penetrate the cell membrane, which changes the physicochemical properties and activities of membrane‐related proteins. This results in reduced transport of calcium and sodium across the immune cell membranes, thereby contributing to rapid immunosuppression.

#### Nongenomic effects mediated bycGCR

Unbound cGCR is a multi‐protein complex, and ligands such as Src, heat shock proteins, and signaling molecules are released from the cGCR multiprotein complex after binding to glucocorticoids, thereby producing rapid effects of immunosuppression.

#### **Non‐genomic effects mediated by membrane‐bound** **GCR** **(** **mGCR** **)**

mGCRs exist on the surfaces of immune cells, and mGCR‐mediated nongenomic effects can be generated rapidly upon binding of glucocorticoids to mGCR.

## **Effects of** **GCS** **on the immune system**

GCS exert effects on a variety of primary and secondary inflammatory cells, as shown in Table 1.^6^ GCS reduce the activation, proliferation, differentiation, and survival of various inflammatory cells, including T lymphocytes and macrophages. GCS also promote cell apoptosis by regulating the production of cytokines, especially the apoptosis of immature and activated T lymphocytes. GCS does not cause significant acute changes in the number of circulating B lymphocyte,^7^ and no study has shown that the level of IgM is affected by GCS.^8^, ^9^ GCS can slightly stimulate neutrophils. In addition, GCS can inhibit the production of adhesion molecules, thereby inhibiting the adhesion of endothelial cells to reduce vascular permeability, which results in the reduction of inflammatory exudation. Super‐physiological concentrations of GCS can also inhibit the proliferation of fibroblasts and the secretion of interleukin‐1 (IL‐1) and tumor necrosis factor alpha (TNFa). As a result, GCS show effective anti‐inflammatory effects. It is believed that ICI therapy leads to abnormal activation of T lymphocytes, and the irAEs are mainly due to the immune attack by these activated T lymphocytes, which are confirmed by pathological biopsy in various types of irAEs including myocarditis, pneumonitis, and skin toxicities.^10^, ^11^, ^12^ Therefore, GCS therapy is the first‐line treatment for irAEs.

**Table 1:** Effects of glucocorticoids on primary and secondary immune cells^6^

Immune cells	Effects of glucocorticoids
Macrophages/monocytes	↓ Number of circulating cells (↓ myelopoiesis, ↓ release) ↓ Expression of MHC class II molecules and Fc receptors ↓ Synthesis of proinflammatory cytokines (eg, IL‐2,IL‐6, TNF_) and prostaglandins
T cells	↓ Number of circulating cells (redistribution effects) ↓ Production and action of IL‐2 (most important) (Little effect on the secretion of immunoglobulin)
Granulocytes	↓ Number of eosinophils and basophile granulocytes ↑ Number of circulating neutrophils
Endothelial cells	↓ Vessel permeability ↓ Expression of adhesion molecules ↓ Production of IL‐1 and prostaglandins
Fibroblasts	↓ Proliferation ↓ Production of fibronectin and prostaglandins

## Types of GCS

The types of GCS used in clinical settings are shown in Table 2.^6^ According to the half‐life of GCS in plasma, GCS can be divided into short‐, medium‐ and long‐acting categories. Oral GCS are rapidly absorbed within 30 minutes. Prednisone and prednisolone have high oral bioavailability. Overall, 90–95% of cortisol in plasma is combined with plasma albumin resulting in no biological activity, while the remaining 5%–10% of free cortisol retains biological activity. Therefore, the level of plasma albumin can affect both the curative and adverse effects of GCS. For patients with hypoalbuminemia and liver disease, the dosage should be adjusted. When treating grade 3–4 irAEs, the initial treatment usually requires at least 1–2 mg/kg of prednisone, and a total course duration of 4–6 weeks or longer.^13^, ^14^ Medium‐acting GCS, such as prednisone or methylprednisolone, are often selected. Dexamethasone also has strong anti‐inflammatory effects, but its half‐life is long, and it is difficult to control the adverse effects when it is used for a long time. Therefore, dexamethasone is generally not selected to treat irAEs.

**Table 2:** Types of hormones and their pharmacokinetic characteristics

		Equivalent dose (mg)	Relative glucocorticoid activity	Relative mineralocorticoid activity	Protein binding capacity	Plasma half‐life (h)	Biological half‐life (h)
Short‐acting	Cortisone	25	0.8	0.8	−	0.5	8–12
	Hydrocortisone	20	1	1	++++	1.5–2	8–12
Medium‐acting	Methylprednisolone	4	5	0.5	−	>3.5	18–36
	Prednisolone	5	4	0.6	++	2.1–3.5	18–36
	Prednisone	5	4	0.6	+++	3.4–3.8	18–36
	Triamcinolone	4	5	0	++	2– > 5	18–36
Long‐acting	Dexamethasone	0.75	20–30	0	++	3–4.5	36–54
	Betamethasone	0.6	20–30	0	++	3–5	36–54

−: No；++: High; +++: High to very high; ++++: Extremely high.

## Principles of clinical application of GCS in irAEs

As mentioned earlier, GCS act via genomic and nongenomic mechanisms, and different doses of GCS can induce therapeutic effects and adverse effects of different strengths and speeds. In the treatment of rheumatic immune diseases, the dose of GCS is determined according to the location of tissue damage, severity, how rapidly the inflammation is developing, and the potential life‐threatening risks of the disease. The grades of dosage of GCS and their clinical applications in rheumatic diseases are shown in Table 3. Large doses of GCS are suitable for rheumatic diseases with more acute onset and severe illness. However, the larger the dose of GCS, the stronger its genomic and nongenomic effects, which can result in faster and stronger immunosuppressive effects. Therefore, in cases of life‐threatening rheumatic diseases, an ultra‐large dose or pulse dose of GCS can be considered. However, larger doses of GCS are also accompanied by greater adverse effects, especially in the case of pulse doses, which often cause early, severe or rare adverse effects that are not common in lower doses of GCS. The duration of GCS of large doses or pulse dose should be short to control the adverse effects of GCS.

**Table 3:** Classification of dose levels of GCS in rheumatological diseases

Classification	Equivalent dose of prednisone	Clinical application	Genomic actions (receptor saturation)	Nongenomicic actions	Adverse effects
Low dose	≤7.5 mg/day	Maintenance therapy for many rheumatic diseases	+ (<50%)	?	Relatively few
Median dose	>7.5 mg/day to ≤30 mg/day	Initial treatment for primary chronic rheumatic diseases	++ (>50% to <100%)	(+)	Dose‐dependent, more if used for a long time
High dose	>30 mg to ≤100 mg/day	Initial treatment for subacute rheumatic diseases	++ (almost 100%)	+	Cannot be used for long‐term treatment because of serious adverse effects
Very high dose	>100 mg/day	Initial treatment for acute and/or potentially life‐threatening exacerbations of rheumatic diseases	+++ (almost 100%)	++	Cannot be used for long‐term treatment because of very serious adverse effects
Pulse therapy	≥250 mg/day for one or few days	For particularly severe and/or potentially life threatening forms of rheumatic diseases	+++ (100%)	+++	More early and serious adverse effects, and more unusual adverse effects

Timely use of GCS is critical for the treatment of irAEs. The strategies used for GCS treatment in irAEs can also be applied as the treatment approach in rheumatic diseases. The dosage of GCS is usually selected according to clinical factors such as organs involved, severity, and the course of irAEs (see Table 4).^14^ Pulse GCS is recommended for major organ injuries with urgent disease course and life‐threatening symptoms, such as myocardial toxicity and neurotoxicity, and especially explosive myocarditis or transverse myelitis. These irAEs have a rapid course of disease, rapid progress, and high mortality, and it is essential to stop the immune injury as soon and as much as possible. For grade 3 to 4 irAEs affecting most other organs, such as colitis, dermatitis, and pneumonitis, high‐dose GCS therapy (prednisone 1–2 mg/kg/day or its equivalent dose) is recommended, taking into account both curative and adverse effects. For irAEs involving the endocrine glands, the clinical manifestations of irAEs involving the endocrine glands are mainly due to insufficient hormone secretion by the endocrine glands, and systemic inflammatory reactions rarely exist. Only physiological dose of GCS (hydrocortisone 15mg to 20mg per day) was needed as supplementary. In terms of dosage forms, injuries to the digestive system such as colitis usually affect the absorption of oral GCS, and thus intravenous GCS is recommended when gastrointestinal tract injury is present. Skin injury can also be treated with topical GCS. In terms of the GCS treatment course, irAEs generally require more than four weeks, and sometimes 6–8 weeks or longer, especially for pneumonitis and hepatitis that are prone to relapse.

**Table 4:** Principles for the use of GCS recommended by NCCN guidelines

irAE	Grade	Type of hormone	Initial dose (mg/kg/day)
Dermatologic	G2 G3‐4	Prednisone Prednisone/Methylprednisolone	0.5–1 0.5–1–2
Diarrhea/colitis	G2 G3‐4	Prednisone/Methylprednisolone Methylprednisolone	1 2
Hepatic toxicity	G2 G3 G4	Prednisone Prednisone/Methylprednisolone Methylprednisolone	0.5–1 1–2 2
Pancreatitis	G2 G3 ~ 4	Prednisone/Methylprednisolone Prednisone/Methylprednisolone	0.5–1 1–2
Pulmonary	G2 G3‐4	Prednisone/Methylprednisolone Prednisone/Methylprednisolone	1–2 1–2
Renal	G2 G3‐4	Prednisone Prednisone/Methylprednisolone	1–2 1–2
Musculoskeletal	G2 G3‐4	Prednisone	0.5 1
Guillain‐Barré syndrome Transverse myelitis	G2/G3/G4	Methylprednisolone	1000 mg/day
Cardiovascular	G3 G4	Methylprednisolone	1000 mg/day

Routine prophylactic use of GCS is not recommended.

## Management of adverse effects of GCS

Long‐term GCS treatment is associated with a series of adverse effects that can affect multiple systems. Musculoskeletal system adverse effects include osteoporosis, osteonecrosis, and steroid myopathy. Digestive system adverse effects include peptic ulcers, pancreatitis, and fatty liver. Cardiovascular system adverse effects include hypertension, premature arteriosclerosis, and arrhythmia. Endocrine system adverse effects include hyperglycemia, lipid metabolism disorder, water‐sodium retention, electrolyte disorder, hypothalamic‐pituitary‐adrenal (HPA) axis inhibition, gonadal inhibition, increased appetite, and weight gain. Mental and behavioral abnormalities include insomnia, emotional instability, and cognitive impairment. The risk of opportunistic infection (pathogens including fungus, tuberculosis and Pneumocystis carinii) is also present. Other adverse effects include cataracts, glaucoma, and skin changes such as acne, purple lines, skin fragility, ecchymosis, hirsutism, and wound‐healing disorders.^6^ However, the risks of various adverse effects vary according to the dose and course of GCS. Low‐dose of GCS is associated with a low risk of infection, but the risk can increase in patients with other underlying complications, such as uncontrolled diabetes mellitus or hypoalbuminemia. Clinically, the time of occurrence of various GCS‐related adverse effects is different. Psychiatric symptoms may occur after the first dosage of GCS, while water‐sodium retention, electrolyte disturbance, heart rate increase, and blood pressure increase are likely to occur during the first three days of GCS treatment. Blood sugar may begin to increase after one week, and opportunistic infections usually occur after three weeks of GCS therapy, but may occur earlier in cases of pulse GCS treatment. Long‐term use of GCS for more than two months may lead to the occurrence of osteoporosis, and more than three months of GCS therapy may cause Cushing's disease, while adrenocortical insufficiency occurs after a longer course of GCS treatment.

It is important to note that an appropriate dose of GCS should be selected as the initial treatment of irAEs, and the course of large‐dose GCS treatment should be controlled within 2–3 weeks. During GCS therapy, blood pressure, blood glucose, and electrolyte levels should be routinely monitored, and the patient should be observed for presence of opportunistic infections.

Prophylactic medication can be used during GCS therapy. Proton pump inhibitors or H2 blockers can be used during high‐dose GCS therapy, especially pulse GCS therapy, or for patients at high risk of gastrointestinal hemorrhage. For patients whose prednisone dose is more than 20 mg per day and course duration is four weeks or more, prophylactic anti‐Pneumocystis carinii therapy should be considered. Supplementation with vitamin D and calcium can be given at the same time.^14^ In addition, patients should avoid contact with infected patients or infection sources, and attention to diet should be given to prevent substantial weight gain.^13^

The risk of infection after GCS therapy is associated with a decrease in blood CD4+ T cell count. It is necessary to monitor the changes in patients' symptoms in order to detect opportunistic infections. For patients with opportunistic infections, timely empirical antibiotic therapy should be used, and etiological evidence should be actively sought so that anti‐infection treatment can be adjusted.

## Disclosure

The authors declare that they have no potential conflicts of interest, financial interests, relationships and affiliations relevant to the subject of their manuscript.
